# Beyond Euglycemia: Case Studies Using Continuous Glucose Monitoring in Elite Athletes Without Diabetes During Record Athletic Events

**DOI:** 10.3390/s26051624

**Published:** 2026-03-05

**Authors:** Kristina Skroce, Lauren V. Turner, Andrea Zignoli, David J. Lipman, Howard C. Zisser, Michael C. Riddell

**Affiliations:** 1 Faculty of Medicine, University of Rijeka, 51000 Rijeka, Croatia; kristina.skroce@student.uniri.hr; 2 Hospital for Medical Rehabilitation of Heart and Lung Diseases and Rheumatism Thalassotherapia-Opatija, 51410 Opatija, Croatia; 3 School of Kinesiology and Health Science, Muscle Health Research Centre, York University, Toronto, ON M3J 1P3, Canada; turnel4@yorku.ca; 4 Department of Industrial Engineering, University of Trento, 38123 Trento, Italy; andrea.zignoli@unitn.it; 5 Independent Researcher, London, UK; davidjacklipman@gmail.com; 6 Independent Researcher, Santa Barbara, CA, USA; hzisser@gmail.com

**Keywords:** continuous glucose monitoring, exercise, glycemia, hyperglycemia

## Abstract

**Highlights:**

**What are the main findings?**

- CGM revealed markedly different glucose responses in elite athletes without diabetes depending on exercise modality, intensity, and fueling strategy, with both hypoglycemic and hyperglycemic excursions occurring well outside conventional euglycemic ranges.
- Extreme and discipline-specific physiological stressors, including intermittent ultra-endurance cycling, uninterrupted prolonged climbing, and hypoxic breath-hold diving, produced distinct and reproducible CGM patterns despite preserving metabolic health.

**What are the implications of the main findings?**

- CGM metrics and clinical glycemic thresholds require context-specific interpretation in elite sport, as transient deviations from euglycemia may reflect adaptive physiological responses rather than metabolic dysfunction.
- These findings support the development of sport- and task-specific CGM benchmarks and highlight the need to integrate CGM data with exercise context, fueling practices, and performance demands when applied in high-performance settings.

**Abstract:**

Glucose data regarding extreme elite performances in athletes without diabetes remains limited. The purpose is to characterize continuous glucose monitoring (CGM) responses in elite athletes across distinct high-performance contexts. This descriptive case series includes three separate elite athletes who used a CGM during their respective sporting events. The first is an ultra-endurance relay cycling world-record performance (Race Across the West, RAW), the second is a continuous high-intensity Everesting Challenge cycling record attempt, and the third is a maximal constant-weight no-fins breath-hold depth dive performed in international competition. Glycemic outcomes, as measured by CGM, included mean, maximum, and minimum glucose, glucose standard deviation (SD), and the percentage of time in tight glucose range (TITR: 70–140 mg/dL; 3.9–7.8 mmol/L), time below range (TBR: <70 mg/dL; <3.9 mmol/L), and time above range (TAR140: >140 mg/dL; >7.8 mmol/L). Other performance data, including peak power, heart rate, and lactate, are also provided where available. During the RAW challenge lasting 44 h and 20 min, mean glucose was 91 ± 23.2 mg/dL (mean ± SD) with 9.15% TBR and 35.58% TITR during cycling and 115 ± 24.7 mg/dL with 9.11% TBR and 43.16% TITR during resting periods. In contrast, the Everesting Challenge cycling record attempt demonstrated a persistently elevated glucose profile (160 ± 5.7 mg/dL), minimal variability (CV 3.5%), and 100% TAR140. Following the maximal breath-hold depth dive, interstitial glucose was 100% TAR140 during recovery (187 ± 18.5 mg/dL), alongside marked elevations in blood lactate concentrations (peak 13.4 mmol/L). The series of case studies demonstrate that substantial deviations from traditional euglycemic ranges are common during elite performance in athletes without diabetes. Interpretation of CGM data in athletic settings should therefore be performance- and context-specific rather than based on clinical glycemic thresholds.

## 1. Introduction

Continuous glucose monitoring (CGM) systems, originally developed to help in individuals with diabetes better manage their insulin therapy and glycemia, are increasingly being adopted by professional athletes without diabetes as a “biofeedback tool” for glycemia regulation [1,2]. Emerging evidence suggests that CGM may help athletes without diabetes identify nutritional and training strategies help maintain stable blood glucose levels during exercise bouts by mitigating reactive hypoglycemia and by facilitating the detection of overnight hypoglycemia during intensified training periods [3]. The utility of CGM has been further explored in tracking glycemic responses to endurance training and subsequent recovery phases, offering insight into exercise-induced metabolic shifts caused by under- or over-fueling [4].

Hypoglycemia has been shown to impair exercise performance, with early research linking the onset of fatigue during prolonged physical activity to declining blood glucose concentrations [5,6]. This underscores the critical importance of maintaining euglycemia during endurance exercise. Conversely, chronically elevated blood glucose levels in the general population are associated with adverse health outcomes, including an increased risk of pre-diabetes and/or cardiovascular disease [7]. Excessive hyperglycemia may also be detrimental for the physiological adaptations to exercise training [8]. However, current evidence does not support a clear link between transient, exercise-induced hyperglycemia and long-term health risks in endurance-trained individuals.

In individuals without diabetes, interstitial glucose levels typically range between 70 and 140 mg/dL (3.9–7.8 mmol/L) > 90% of the time, the so-called “time in tight range” (TITR). A multicenter prospective study involving 153 healthy participants aged 7 to 80 years reported a mean 24 h glucose concentration of 99 ± 7 mg/dL (5.5 ± 0.4 mmol/L), with the median percentage of time spent within the TITR was 96% [9]. However, studies published in elite and semi-elite athletes show different glucose levels than those of healthy but non-active controls. For example, daily TITR was 91% in elite para cyclists [10], 93% in professional female cycling team [11] and 89% in well-trained cyclists [12]. Limited data indicates that elite athletes may experience greater glycemic variability than non-athletic controls, including spending more time in both the hypoglycemic (<70 mg/dL; TBR) and hyperglycemic (>140 mg/dL; TAR_140_) ranges [12]. In the present article, we highlight three case studies of elite athletes who completed extreme, high-performance events while using CGM, along with some other wearable sensors that helped to quantify the exercise events. One athlete completed a 3-day cycling race across the western United States (Race Across the West; https://www.raceacrossthewest.org/ accessed on 2 March 2026), another completed a timed cycling event with an elevation gain equivalent to that of Mount Everest (8848 m), and the third completed a constant-weight no-fins breath-hold depth dive (93 m) performed in international competition. Our objective is to use these case studies to characterize glucose dynamics in response to exceptional and unique sporting events.

## 2. Materials and Methods

### 2.1. CGM Device Information and Use

Interstitial glucose was measured using a CGM biosensor (Abbott Libre Sense Glucose Sport Biosensor, Abbott Diabetes Care, Alameda, CA, USA) which recorded glucose values (minimum to maximum range 54–200 mg/dL [3.0–11.1 mmol/L]) at a 1 min sampling frequency via an integrated surface recorder/transmitter that communicated in real time with a smartphone application (Supersapiens Inc., TT1 Products, Atlanta, GA, USA) using Bluetooth technology.

The Abbott Libre Sense Glucose Sport Biosensor uses enzymatic electrochemical sensing of interstitial glucose via a subcutaneous filament. The system is factory-calibrated and does not require capillary blood glucose calibration during wear. The sensor is approved for continuous use for up to 14 days, and all events included in the present manuscript were conducted within this validated wear period.

The Libre Sense biosensor is based on the same core sensing technology as the FreeStyle Libre system, which has demonstrated mean absolute relative differences (MARD) of approximately 9–14% compared with reference glucose measurements in clinical and ambulatory settings [13,14]. In an exercise-specific validation study involving well-trained male athletes, the system demonstrated a MARD < 15%. Bland–Altman analysis showed a mean bias of 0.40 ± 14 mg/dL, with 95% limits of agreement from −26 to +27 mg/dL across varying exercise intensities, supporting acceptable analytical performance under exercise conditions [15].

In athletic settings, such as those presented in each case study, reliable signal acquisition depends primarily on maintaining sensor stability and preserving the sensor–skin interface during movement and environmental stress. Sensors were placed on the athletes’ posterior upper arm according to manufacturer recommendations to minimize mechanical compression and friction. The skin was cleaned and fully dried prior to application, and an overpatch was used to reduce detachment risk due to sweating or shear forces. Sweat does not directly interfere with the enzymatic glucose detection mechanism.

Glucose values are recorded at 1 min intervals and transmitted via Bluetooth to a compatible smartphone application. In the case of a temporary Bluetooth interruption, data are automatically synchronized once connectivity is restored. If the smartphone is out of range, the sensor stores glucose values at 15 min intervals for up to 8 h and uploads them upon reconnection, thereby minimizing data loss.

Across all three cases, CGM was used extensively during training to evaluate glucose responses and refine nutritional strategies. During the documented events, however, CGM data was not readily available to the Everesting or free-diving athletes. The exception was the Race Across the West case, in which real-time CGM data were available. However, the athlete reported that the nutritional strategy had been predefined during prior course-specific rehearsals using CGM-guided testing, and no in-event adjustments were to be made based on live glucose readings. Accordingly, in all three documented cases, CGM data were recorded for post-event analysis only and were not used as a real-time biofeedback tool by the athletes to guide performance-related adjustments.

The Supersapiens App User Agreement and Privacy Policy accepted by the user stated that anonymized data can be used for public and/or research purposes. Moreover, all athletes highlighted in the case studies additionally consented to data sharing.

### 2.2. Data Handling

CGM data for each case-study was examined for descriptive statistics only. The CGM data review allowed for the identification of any potential artefactual segments or missing data, which were excluded from the analysis. CGM readings were truncated at the lower (54 mg/dL) and upper (200 mg/dL) CGM-detection limits. All data is presented as mean ± standard deviation (SD).

CGM data reported over the time-period of each presented case includes (1) mean glucose, the average glucose value across the specified event duration; (2) maximum glucose, the highest recorded glucose value during the event; (3) minimum glucose, the lowest recorded glucose value during the event; (4) glucose standard deviation (SD), reflecting the absolute variability of glucose values around the mean; (5) glucose coefficient of variation (%CV), calculated as (SD ± mean glucose) × 100 to express relative glycemic variability; percent time in tight glucose range (TITR: 70–140 mg/dL; 3.9–7.8 mmol/L), defined as the proportion of total event time during which glucose values fell within this range; (7) percent time below range (TBR: <70 mg/dL; <3.9 mmol/L), defined as the proportion of event time with glucose values below this threshold; and (8) percent time above range (TAR140: >140 mg/dL; >7.8 mmol/L), defined as the proportion of event time with glucose values exceeding this threshold. In the context of the presented cases, these metrics should be interpreted descriptively as markers of exercise- and fueling-specific glycemic dynamics rather than as clinical indicators of glycemic control.

## 3. Results

### 3.1. Case Study 1: Professional Cyclist: Race Across the West: World Record

#### 3.1.1. Participant

The participant is a 26-year-old professional level male ultra-endurance cyclist (body mass 60 kg, height 185 cm) who trained and raced regularly in the three years preceding the present event and had a specific focus on ultra-cycling world records. In the two years before the event, he established three world records: a 6 h time trial (248.36 km) and a 100 km (02:23:00 h:min:s) and 200 km (04:48:00 h:min:s) distance race. As such, at the time of the present event, he was an experienced ultra-endurance athlete accustomed to prolonged competition, high training volumes, and structured race-day fueling strategies.

#### 3.1.2. Sport Event Details

The Race Across the West (RAW, www.raceacrossthewest.org) is an ultra-endurance cycling event from Oceanside, California, to Durango, Colorado, following the opening segment of the Race Across America (RAAM) route. In the record-setting year, the RAW course was 825.65 miles (≈1329 km) long and featured a substantial total elevation gain (12,700 m). Athletes may compete solo or in relay teams of two or four riders. In this case study, the athlete competed in the two-person relay division, successfully winning the event and setting a new course record with a total finishing time of 44 h and 20 min. This edition of RAW started in Oceanside on 13 June at 12:03 pm (noon) local time and finished in Durango at 9:23 am local time on 15 June, normally covering up to three calendar days (13–15 June). The racing team employed a rotation-based pacing strategy, with athletes alternating cycling efforts roughly every 20–40 min. This approach allowed each rider brief recovery periods between high intensity cycling bouts.

#### 3.1.3. CGM and Performance Data

**Figure 1:**
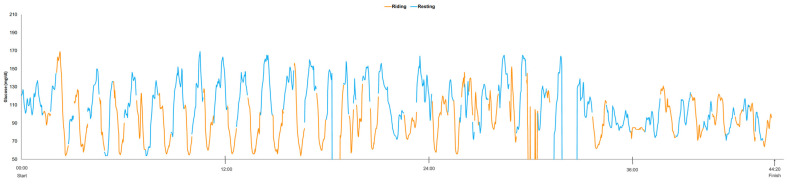
Interstitial glucose dynamics during the Race Across the West ultra-endurance relay cycling event. CGM data are shown across the full duration of the two-person RAW event (44 h 20 min). Glucose values are color-coded according to activity state: orange segments represent glucose values recorded during active cycling, whereas blue segments represent glucose values recorded during off-bike recovery periods (i.e., while the other team member was undergoing active cycling). A discontinuity in the CGM trace is shown at ~18 h into the race and intermittently between ~28 and 31 h, indicating a total of ~3 h 30 min of missing CGM data, which were excluded from all quantitative analyses.

Figure 1 illustrates the athletes CGM trace during the entire RAW event encompassing both riding and rest periods, lasting a total of 44 h and 20 min in duration. Of note, the athlete’s CGM profile shows both off-bike (blue lines) and on-bike (orange lines) CGM values. The interstitial glucose trace demonstrates a highly dynamic pattern that appears to be aligned with these alternating periods of rest and exercise, with repeated glucose rises during rest periods (blue) and declines during cycling periods (orange).

Across the race, mean interstitial glucose during cycling segments was 91 ± 23.2 mg/dL, compared with 115 ± 24.7 mg/dL during resting periods (see Table 1). The minimum and maximum glucose values recorded were 54 mg/dL and 169 mg/dL, respectively, during both cycling and resting periods combined. TBR accounted for 9.15% of cycling time and 1.23% of resting time. Accordingly, TITR was lower during cycling relative to resting time (35.58% vs. 43.16%). TAR_140_ was 1.77% during cycling and was more frequently observed during resting periods, accounting for 9.11%.

Performance parameters, including heart rate (HR, bpm), power output (W), cadence (rpm), speed (km/h), distance (km), and elevation gain (m), were continuously assessed using a Garmin Edge 530 (Garmin Ltd., Olathe, KS, USA) cycling computer. Mean power output and heart rate during cycling segments were 210 W (range: 0–531 W) and 142 bpm (range: 82–167 bpm), respectively.

#### 3.1.4. Carbohydrate Intake and Nutritional Strategy

Total carbohydrate consumption throughout the race is summarized in Table 2. Across the full duration of the event, carbohydrate intake averaged 58 g/h, although intake was not evenly distributed across race days. For example, on day 1 after race start (starting at ~12 PM), intake was highest at approximately 92 g/h, followed by a reduction to 53 g/h by day 2 and 31 g/h by day 3. Carbohydrate sources included carbohydrate drink mixes, sports gels with varying glucose-to-fructose ratios, solid foods (sports bars, oats, and sandwiches), and liquid nutritional supplements. In addition to carbohydrate-based fueling, the athlete incorporated exogenous ketones (R-1,3-butanediol) during the race as part of his nutritional strategy. Electrolyte intake was provided via drink mixes and supplemental electrolyte products. Notably, all nutritional intake occurred exclusively during the off-bike recovery periods, with no feeding during active cycling.

### 3.2. Case Study 2: Professional Cyclist: Everesting Challenge

#### 3.2.1. Participant

The participant is a 34-year-old male former professional road cyclist (body mass 70 kg, height 182 cm) who previously competed at the UCI (Union Cycliste Internationale, world cycling governing body) Continental level. He has an extensive background in elite endurance cycling, having raced internationally across stage races and one-day events during his professional career. Following his professional road racing career, the athlete transitioned toward record-oriented endurance performances. In the year preceding the present event, he completed the same course in an official time of 07:04:41 h:min:s, providing a well-documented personal benchmark against which the current record attempt can be contextualized.

#### 3.2.2. Sport Event Details

The Everesting Challenge (www.everesting.com) is a cycling, running, hiking, stair climbing, or skiing challenge which consists of repeatedly ascending and descending a single climb (the competitor can select their location) in one continuous, uninterrupted effort until a cumulative elevation gain equivalent to the height of Mount Everest (8848 m) is achieved. Athletes may select any route, provided the same climb is used for all repetitions. At the time of the highlighted athletes challenge attempt, more than 30,000 successful Everesting completions had been recorded globally across 107 countries, highlighting the growing popularity of the challenge.

**Figure 2:**
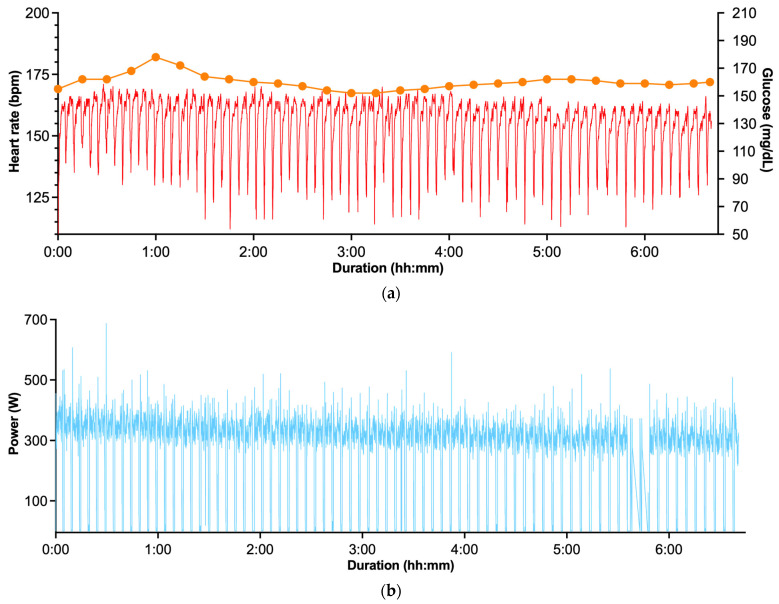

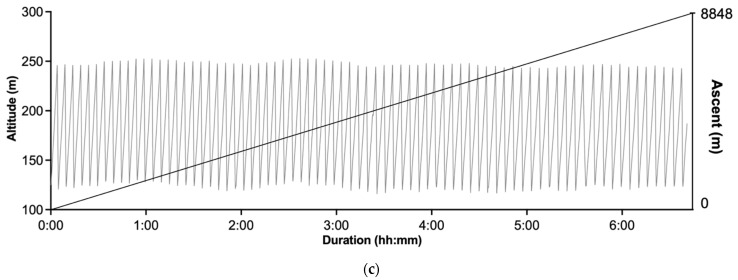
The distinct panels show continuous glucose monitoring and performance data during an “Everesting” record attempt, with panels denoting (**a**) time course of interstitial glucose concentration (CGM) (orange) across the entire “Everesting” cycling effort (i.e., cycling climbing elevation gain equivalent to the height of Mt Everest), illustrating a sustained TAR profile throughout the climb repetitions and time-aligned heart rate (red); (**b**) power output and across the entire Everesting effort; and (**c**) course profile and elevation gain of the Everesting attempt performed on the Mamore Gap (Republic of Ireland). The athlete completed repeated ascents and descents of an 810 m road segment, accumulating a total vertical gain of 8848 m in just under 7 h. Glucose data were recorded at 15 min intervals during periods when the smartphone was out of range of the sensor (during the record attempt), as CGM devices store data at a 15 min sampling frequency for up to 8 h when not actively connected. Notes: TAR140 = time > 140 mg/dL.

In the present case study, the athlete completed the Everesting Challenge by cycling in 06:40:54 h:min:s, setting a new performance benchmark. The record-setting attempt was performed on the Mamore Gap in Republic of Ireland, using an 810 m road segment with an average gradient of 14.2%, corresponding to approximately 117 m of elevation gain per ascent. The athlete completed 76 repeated ascents and descents of this segment, accumulating a total distance of 123.12 km over the course of the effort. CGM and performance data for the Everesting Challenge record attempt are shown in Figure 2a–c.

#### 3.2.3. CGM and Performance Data

Mean glucose concentration during the attempt was 160 ± 5.7 mg/dL, with observed values ranging from 151 (minimum) to 178 mg/dL (maximum). Glucose variability was low, with a coefficient of variation of 3.5%. The athlete’s percent time in range values over the course of the Everesting Challenge were reflective of this limited variability, with 0% of the time in TITR or TBR, and had 100% TAR140 during the record attempt (see Table 1). Performance parameters, including heart rate (HR, bpm), power output (W), cadence (rpm), speed (km·h^−1^), distance (km), and elevation gain (m), were continuously assessed using a Wahoo ELEMNT BOLT (Wahoo Fitness, Atlanta, GA, USA) cycling computer. Across the entire ride, mean power output was 296 W (range: 0–688 W), with a corresponding average heart rate of 154 bpm (range: 107–171 bpm).

#### 3.2.4. Carbohydrate Intake and Nutritional Strategy

Total nutritional intake during the Everesting Challenge is summarized in Table 3. Mean carbohydrate intake across the event was approximately 149 g/h. Carbohydrate intake was delivered primarily via liquid and semi-solid sources, enabling frequent ingestion while maintaining uninterrupted cycling.

### 3.3. Case Study 3: Breath Hold Deep Dive

#### 3.3.1. Participant

The athlete is 31-year-old male (body mass 84 kg, height 182 cm) who had more than three years of competitive breath-hold diving experience at the time of data collection. His training program included year-round structured preparation. Across the season, the athlete accumulated approximately 12–20 h of total training per week, with weekly volume varying according to training phase (base, pre-competition, and competition periods). Apnea-specific training, which included pool and open-water breath-hold sessions, dry static and dynamic apnea exercises, equalization practice, and depth-technique drills, accounted for approximately 6–10 h/week during the pre-competition and competition phases and 4–6 h/week during base training. General physical training aimed at supporting apnea performance (aerobic endurance, resistance training, mobility, and mixed conditioning) accounted for an additional ~6–10 h/week with slightly higher volumes during the base phase. Overall, approximately 40–60% of total weekly training time was devoted specifically to apnea-related practice in the weeks leading up to competition.

#### 3.3.2. Sport Event Details

Data for this case study were collected during the Adriatic Freediving Trophy, an international competition held in Krk, Croatia, in September 2022, sanctioned by AIDA International (https://www.aidainternational.org) and CMAS (Confédération Mondiale des Activités Subaquatiques www.cmas.org). The athlete competed in the constant weight without fins (CNF) discipline. During this competition, the athlete achieved an in-competition CMAS (Confédération Mondiale des Activités Subaquatiques) World Record dive to 93 m.

The competitive 93 m dive consisted of a single uninterrupted breath-hold immersion with a total duration of 3 min 55 s. The first post-dive physiological assessment (sample 01, S01) was performed as soon as feasible following surfacing, approximately 3:00 min upon surfacing. This interval reflected the combined duration of the dive itself and the time required for surfacing procedures and transfer back to the support boat. Capillary lactate, heart rate (HR) and peripheral oxygen saturation (SpO_2_) were assessed at baseline, immediately post-dive (03:00 min from the end-dive, the time required for surfacing procedures and transfer back to the support boat), and every 5 min thereafter up to 1 h 35 min post surfacing (S19). Capillary blood glucose measurements were obtained on-site by the investigating team during the pre- and post-dive periods to verify concordance with CGM-derived interstitial glucose values. Due to agreement, these data are not presented separately.

#### 3.3.3. CGM and Other Physiologic and Performance-Related Data

CGM data were collected continuously before warmup, during the warmup, and after the CNF dive. Baseline capillary lactate (La-, mmol/L), heart rate (HR, bpm) and peripheral oxygen saturation (SpO_2_, %) were recorded prior to water entry and every 5 min after the CNF dive. Additionally, and during the warm-up period, CGM signal acquisition continued uninterrupted as the receiver smartphone remained on the support boat within Bluetooth range. No other physiological measures could be obtained during this phase as the athlete was in the water warming up.

**Figure 3:**
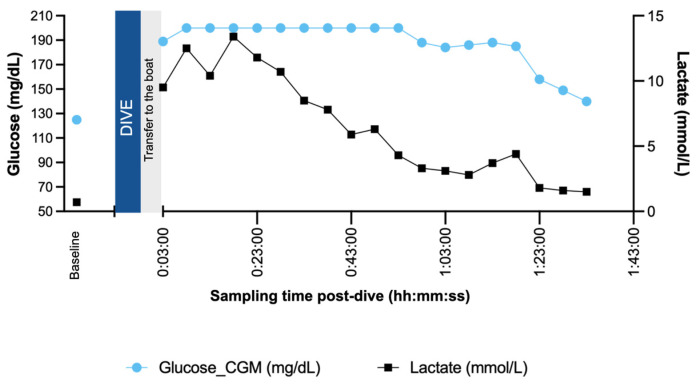
Interstitial glucose and capillary lactate responses following a maximal breath-hold depth dive. Continuous glucose monitoring (CGM) and capillary lactate concentrations are shown across baseline, immediate post-dive, and recovery following a constant-weight without fins (CNF) dive to 93 m. CGM data were recorded before the dive and resumed immediately after surfacing, while lactate was measured at baseline, as soon as feasible post-dive, and at 5 min intervals during recovery. The dive period (3 min 55 s) is indicated on the x-axis. The blue CGM line represents glucose values collected outside of exercise, i.e., prior to and following the dive.

During the dive itself, the CGM signal was “blocked” because of connectivity loss of the sensor to smartphone application, and the first valid value was obtained upon surfacing and transferring the diver to the boat. Across the full post-dive observation period, interstitial glucose concentrations remained consistently above the euglycemic threshold. Time-in-range analysis demonstrated that the athlete spent 0% of monitored TBR and 0% within the TITR, with 100% TAR140 post record attempt. Mean CGM glucose during the post-dive recovery phase was 187 ± 18.5 mg/dL, with a progressive decline observed over the recovery period, returning toward baseline values by the end of monitoring (Figure 3). CGM levels stayed above the 200 mg/dL for 50 min in recovery.

Capillary lactate concentration increased markedly following the dive. Baseline lactate was 0.7 mmol/L, rising to 9.5 mmol/L at S01 and peaking at 13.4 mmol/L during early recovery. Mean post-dive lactate concentration was 6.8 ± 4.0 mmol/L, with values gradually declining toward baseline over the subsequent recovery period. Heart rate (HR) increased from a baseline value of 65 bpm to 92 bpm at S01 and remained moderately elevated during recovery. Mean post-dive HR was 101.3 ± 8.8 bpm, with values stabilizing over time. Peripheral oxygen saturation (SpO_2_) showed a pronounced reduction immediately post-dive (87% at S01), followed by progressive normalization during recovery. Mean post-dive SpO_2_ was 93.8 ± 3.5%.

#### 3.3.4. Carbohydrate Intake and Nutritional Strategy

The diver reported fasting 12 h prior to the dive, and no food or beverages were consumed during the recovery phase where data collection took place.

## 4. Discussion

This series of three case studies provides a unique, discipline-spanning description of CGM responses during exceptional real-world athletic performances in elite athletes without diabetes. Intermittent ultra-endurance cycling (case 1) was characterized by marked glucose oscillations and periods of hypoglycemia, while an uninterrupted uphill cycling challenge (case 2) elicited sustained hyperglycemia with minimal glucose variability. In a third case, maximal breath-hold diving with significant hypoxia produced prolonged post-exercise hyperglycemia despite pre-dive fasting, thus illustrating another unique physiologic stress response. Together, these three separate cases demonstrate that interstitial glucose concentrations in elite athletes without diabetes are highly dependent on exercise modality, intensity, and fueling strategy, and may deviate substantially from the conventional tight “euglycemic” range (i.e., 70–140 mg/dL).

In the Race Across the West case, CGM revealed pronounced glucose oscillations tightly coupled to alternating cycling and resting periods, perhaps because carbohydrate intake was restricted exclusively to off-bike periods that occurred every 20–40 min. Mean interstitial glucose level, as well as percentage TITR, were lower during cycling (91 ± 23.2 mg/dL, 35.58%) than resting (115 ± 24.7 mg/dL, 43.16%); more hypoglycemic exposure (TBR = 9.15% vs. 1.23%) and lower hyperglycemic (TAR_140_) excursions (1.77% vs. 9.11%) were observed during cycling compared to resting.

The observed increase in TBR during cycling segments, despite regular carbohydrate availability during rest periods, may be partially explained by the balance between glucose delivery and utilization during exercise. Carbohydrate-containing beverages results in increased glucose availability for exercise and can be associated with elevations in glycemia while exercise increases glucose disposal rate [16,17,18]. The occurrence of more TBR during cycling may also reflect exercise-associated reactive hypoglycemia, which can occur if carbohydrates are consumed during rest before exercise since they can increase insulin secretion which can then result in a greater drop in glucose once the muscle start exercising [19]. Importantly, Zignoli et al. reported that reactive hypoglycemia is typically “a transient event observed within the first 30 min after exercise commencement” and that its occurrence depends strongly on individual susceptibility and timing of food ingestion [3]. In the present relay format, repeated transitions between rest and high-intensity cycling may repeatedly recreate this metabolic scenario, explaining the recurrent TBR observed despite adequate carbohydrate intake during rest periods.

Moreover, the observed decline in glucose during the cycling bouts may have had performance-related implications. Previous laboratory-based research in healthy participants has demonstrated that greater circulating glucose availability can help preserve neuromuscular function, including maximal voluntary contraction torque and voluntary activation [6], suggesting that reduced glucose availability could plausibly impair performance. However, this relationship cannot be confirmed in the present case, as synchronized minute-by-minute glucose and power output data were not available. Additionally, elevations in perceived exertion have been reported in association with reduced glucose availability [20], but subjective perceptual measures were not collected in the current case. Nevertheless, the athlete had extensively rehearsed the event and refined nutritional strategies using CGM and performance feedback during training, which may have mitigated potential performance decrements. Future studies incorporating synchronized physiological, perceptual, and performance metrics would help clarify these relationships.

Throughout the uninterrupted Everesting Challenge uphill-dominant effort, interstitial glucose remained consistently elevated, with 100% of monitored time spent above 140 mg/dL, minimal glycemic variability, and no hypoglycemia. The increases in glycemia with some intense activities appear related, at least in part, to elevations in circulation catecholamines that increase hepatic glucose production beyond what can be taken up by contracting skeletal muscles [21,22]. Similarly to what the in-match data shows in elite European football players in a recently published manuscript [23], these observations indicate that substantial elevations in CGM values—above the so-called “normal glycemic range” commonly classified as hyperglycemic in clinical populations—are frequently observed in elite athletic contexts and appear to represent physiological responses to extreme exercise stress rather than pathological glucose dysregulation.

Notably, the athlete achieved a very high carbohydrate intake rate during the challenge (~149 g/h), which likely contributed to the “stress-induced” hyperglycemia. Current nutritional guidelines recommend carbohydrate intakes of up to 90 g/h of multiple transportable carbohydrates during prolonged endurance exercise lasting ≥ 2.5 h [19,24]. However, more recent evidence suggests that higher intakes, approaching or exceeding 120 g/h, may confer additional metabolic and performance benefits in well-trained athletes, provided that multiple transportable carbohydrates are used and gastrointestinal tolerance is preserved [25,26]. The athlete’s tolerance of an intake as high as ~149 g·h^−1^ is likely attributable to individual adaptation, including prior nutritional training, gastrointestinal conditioning, and the specific metabolic demands of this event, and should therefore be interpreted on an individual rather than a generalized basis. Carbohydrate intake was delivered primarily via liquid and semi-solid sources, enabling frequent ingestion while maintaining uninterrupted cycling. This high carbohydrate availability likely contributed to the observed stability in interstitial glucose concentrations and the low glycemic variability recorded throughout the attempt.

The breath-hold deep-dive case revealed a distinct glucose response, characterized by a delayed but sustained rise in interstitial glucose during post-dive recovery, occurring alongside a rapid and pronounced increase in blood lactate concentration. The marked lactate accumulation is most likely a consequence of the diving response, an oxygen-preserving mechanism characterized by peripheral vasoconstriction and reduced tissue perfusion [27]. As previously reported, lactate produced during voluntary breath-hold apnea cannot be readily metabolized due to both limited lactate transport between tissues and to generalized hypoxia, resulting in insufficient oxygen availability for lactate oxidation [28]. Consequently, high lactate levels similar to what occurs with maximal exercise efforts but with minimal skeletal muscle contractions are typically observed immediately after surfacing from apneic dives, consistent with the present findings [28].

One possible interpretation of the delayed glucose rise is increased hepatic glucose production during recovery, potentially driven by lactate conversion to glucose via the Cori cycle once reoxygenation occurs. Following surfacing, normalization of pH and CO_2_ elimination may facilitate hepatic gluconeogenesis, while elevated catecholamine concentrations previously documented after deep apnea dives [29] further stimulate hepatic glucose release and increase the rate of glucose appearance in circulation [30]. Alternatively, elevated interstitial glucose may support lactate clearance by providing additional metabolic flexibility during recovery. Lactate itself can inhibit skeletal muscle glucose uptake, as it serves as an oxidative substrate in muscle tissue [31], which may further contribute to elevated circulating glucose concentrations. Finally, it is also possible that the post-dive rise in glucose and the clearance of lactate represent parallel, but independent, responses to a shared physiological stressor involving hypoxia, hypercapnia, acid–base disturbances, and sympathoadrenal activation. Given the observational nature of this case study, these associations should be interpreted cautiously.

Although extreme hypoxia, peripheral vasoconstriction, and reperfusion during breath-hold diving could theoretically influence interstitial glucose measurements, concurrent capillary plasma glucose measurements obtained on site by the investigating team showed no clinically meaningful deviation from CGM values in the peri-dive period. Nevertheless, as CGM systems have not been specifically validated under apnea-induced hypoxic conditions, these findings should be interpreted with appropriate caution.

Taken together, these three cases illustrate that CGM metrics commonly used in clinical populations (TITR, TAR140, TBR) require context-specific interpretation when applied to elite athletes. While prolonged exposure to hyperglycemia is undesirable in clinical settings, the transient elevations observed here occurred in the setting of extreme performance demands, high carbohydrate intake, and intact insulin sensitivity.

The increasing use of real-time CGM in elite sport raises important questions regarding technological assistance or “doping” and its role in fair sport and competition. Access to CGM data, even during training, may enhance fueling precision and potentially inform pacing strategies, thereby impacting performance. However, at present, CGM is permitted (i.e., is not classified as a banned method) by the World Anti-Doping Agency (WADA). It is important to note, however, that sport-specific regulatory bodies may take differing positions. For instance, the Union Cycliste Internationale (UCI) strictly prohibits the use of CGMs during sanctioned cycling races. As wearable technologies continue to evolve, ongoing discussion is warranted regarding fairness, accessibility, and the boundaries between physiological performance monitoring versus performance augmentation in sport.

The present case series extends this literature by demonstrating that exceptional athletic performances can produce glucose profiles well outside normative ranges without apparent adverse outcomes, reinforcing the need for sport- and context-specific CGM benchmarks. However, this study is limited by its descriptive case-series design, which includes a small number of elite athletes and therefore does not allow for generalization or causal inference. Data were collected in real-world competitive settings, limiting experimental control and precluding standardized measurement of key hormonal and metabolic regulators of glucose homeostasis (e.g., insulin, glucagon, catecholamines). Additionally, interstitial glucose measurements obtained via CGM may be subject to physiological lag relative to blood glucose, particularly during rapid transitions in exercise intensity or hypoxic stress.

## 5. Conclusions

The findings from the three case studies presented in this manuscript highlight that transient deviations from euglycemia are common in elite sport, likely reflecting adaptive physiological responses to extreme metabolic, cardiovascular, and neuroendocrine stress rather than pathological dysregulation. CGM, therefore, represents a powerful tool for contextualizing metabolic demands in high-performance settings, but its interpretation must be grounded in exercise modality, intensity, and fueling strategy. Future research should integrate CGM with hormonal, metabolic, and performance outcomes across larger cohorts to better define optimal glucose ranges for elite athletic performance, rather than relying on thresholds derived from clinical populations.

## Figures and Tables

**Table 1:** Glycemic outcomes across different record events.

Outcome	Everesting	Race Across the West: Exercise	Race Across the West: Recovery	Breath Hold Deep Dive: Recovery
iG mean ± SD (mg/dL)	160 ± 5.7	91 ± 23.2	115 ± 24.7	187 ± 18.5
iG min (mg/dL)	151	54	54	125
iG max (mg/dL)	178	169	169	200
%TBR (<70 mg/dL)	0.0	9.15	1.23	0
%TITR (70–140 mg/dL)	0.0	35.58	43.16	0
%TAR140 (>140 mg/dL)	100.0	1.77	9.11	100
iG min (mg/dL)	160 ± 5.7	91 ± 23.2	115 ± 24.7	187 ± 18.5

Abbreviations: iG mean, mean interstitial glucose; iG min, minimum interstitial glucose; iG max, maximum interstitial glucose; TBR, time below range < 70 mg/dL; TITR, time in tight range 70 to 140 mg/dL; TAR140, time above range > 140 mg/dL.

**Table 2:** Nutritional intake during the Race Across the West event.

Nutritional Product Type	Nutritional Product Information	Servings	CHO/ Serving (gr)	Calories/ Serving (kcal)	Total CHO (gr)	Total Calories (kcal)
Carbohydrate drink ^A^	maltodextrin and fructose	24	30	120	720	2880
Energy gel ^A^	0.8:1 fructose-to-glucose ratio	2	25	100	50	200
Sports shake ^A^	fruit and vegetable mix	6	22	220	132	1320
Energy bar ^A^	oats and rice bar	8	45	225	360	1800
Energy bar ^A^	rice bar	8	50	249	400	1992
Ketones ^A^	(R-1,3-butanediol)	8	0	70	0	560
Energy gel ^A^	2:1 maltodextrin-to-fructose ratio	3	90	360	270	1080
Hydration sport drink ^A^	contains 370 mg sodium	9	22	80	198	720
Electrolyte drink ^A^	100 mg of caffeine/serving	6	11	45	66	270
Electrolyte drink, recovery ^A^	/	2	31	180	62	360
Turkey Sandwich	/	2	45	250	90	500
Banana Milk	/	7	14	80	98	560
Peanut Butter	/	3	6	210	18	630
Oats	/	2.5	51	303	127	757.5
Total					2591	13,629

^A^ indicates commercially available products. Nutritional product information, carbohydrate (CHO) content per serving, and caloric values were taken directly from the manufacturers’ product declarations. Total CHO and total energy intake were calculated based on the number of servings consumed during the event.

**Table 3:** Nutritional intake during the Everesting event.

Nutritional Product Type	Nutritional Product Information	Servings	CHO/ Serving (gr)	Calories/Serving (kcal)	Total CHO (gr)	Total Calories (kcal)
Carbohydrate drink ^A^	1:0.8 maltodextrine-to-fructose ratio	7	80	315	560	2205
Energy gel ^A^	isotonic maltodextrine (33%) gel	6	22	87	132	522
Energy gel with caffeine ^A^	150 mg of caffeine/serving	6	22	87	132	522
Energy bar ^A^	/	84	43	172	172	688
Total					996	3937

^A^ indicates commercially available products. Nutritional product information, carbohydrate (CHO) content per serving, and caloric values were taken directly from the manufacturers’ product declarations. Total CHO and total energy intake were calculated based on the number of servings consumed during the event.

## Data Availability

The data supporting the findings of this study are available from the corresponding author upon reasonable request, in accordance with the participants’ consent and data-sharing agreements.

## Abbreviations

The following abbreviations are used in this manuscript:

CGM	Continuous glucose monitoring
TBR	Time below range (<70 mg/dL; <3.9 mmol/L)
TITR	Time in tight range (70–140 mg/dL; 3.9–7.8 mmol/L)
TAR_140_	Time above range (>140 mg/dL; >7.8 mmol/L)
RAW	Race Across the West
RAAM	Race Across America
HR	Heart rate
iG	Interstitial glucose
CHO	Carbohydrate
UCI	Union Cycliste Internationale
CNF	Constant weight without fins
AIDA	International Association for the Development of Apnea
CMAS	Confédération Mondiale des Activités Subaquatiques
